# Comparison of endocrine therapy and chemotherapy as different systemic treatment modes for metastatic luminal HER2-negative breast cancer patients —A retrospective study

**DOI:** 10.3389/fonc.2022.873570

**Published:** 2022-07-26

**Authors:** Qiuyue Liu, Juan Qiu, Qianrun Lu, Yujin Ma, Shu Fang, Bing Bu, Lihua Song

**Affiliations:** ^1^ Shandong Cancer Hospital and Institute, Shandong Academy of Medical Sciences, Shandong First Medical University, Jinan, China; ^2^ Oncology Department, The Fourth People’s Hospital of Jinan, Jinan, China; ^3^ Department of Breast Medicine, Shandong Cancer Hospital and Institute, Shandong Academy of Medical Sciences, Shandong First Medical University, Jinan, China

**Keywords:** metastatic breast cancer, chemotherapy, endocrine therapy, systemic treatment modes, prognosis

## Abstract

**Purpose:**

The purpose of this study was to evaluate endocrine therapy and chemotherapy for first-line, maintenance, and second-line treatment of hormone receptor-positive HER-2-negative metastatic breast cancer (HR+HER-2-MBC) and the relationship between different treatment options and survival.

**Patients and methods:**

The patients included in this study were all diagnosed with metastatic breast cancer (MBC) at Shandong Cancer Hospital from January 2013 to June 2017. Of the 951 patients with MBC, 307 patients with HR+HER-2-MBC were included in the analysis. The progression-free survival (PFS) and overall survival (OS) of the various treatment modes were evaluated using Kaplan–Meier analysis and the log-rank test. Because of the imbalance in data, we used the synthetic minority oversampling technique (SMOTE) algorithm to oversample the data to increase the balanced amount of data.

**Results:**

This retrospective study included 307 patients with HR+HER-2-MBC; 246 patients (80.13%) and 61 patients (19.87%) were treated with first-line chemotherapy and first-line endocrine therapy, respectively. First-line endocrine therapy was better than first-line chemotherapy in terms of PFS and OS. After adjusting for known prognostic factors, patients receiving first-line chemotherapy had poorer PFS and OS outcomes than patients receiving first-line endocrine therapy. In terms of maintenance treatment, the endocrine therapy-endocrine therapy maintenance mode achieved the best prognosis, followed by the chemotherapy-endocrine therapy maintenance mode and chemotherapy-chemotherapy maintenance mode, and the no-maintenance mode has resulted in the worst prognosis. In terms of first-line/second-line treatment, the endocrine therapy/endocrine therapy mode achieved the best prognosis, while the chemotherapy/chemotherapy mode resulted in the worst prognosis. The chemotherapy/endocrine therapy mode achieved a better prognosis than the endocrine therapy/chemotherapy mode. There were no significant differences in the KI-67 index (<15%/15-30%/≥30%) among the patients receiving first-line treatment modes, maintenance treatment modes, and first-line/second-line treatment modes. There was no statistical evidence in this study to support that the KI-67 index affected survival. However, in the first-line/second-line model, after SMOTE, we could see that KI-67 ≥ 30% had a poor prognosis.

**Conclusions:**

Different treatment modes for HR+HER-2-MBC were analyzed. Endocrine therapy achieved better PFS and OS outcomes than chemotherapy. Endocrine therapy should be the first choice for first-line, maintenance, and second-line treatment of HR+HER-2-MBC.

## 1 Introduction

Breast cancer exceeded lung cancer as the most commonly diagnosed cancer among women in 2020 (1). It is estimated that approximately 20-30% of breast cancer patients have metastases at the time of diagnosis (2, 3). Despite the progress achieved in breast cancer management, 20-30% of early-stage breast cancer patients experience recurrence and distant metastasis (4, 5). Thus, advanced breast cancer poses a serious threat to women’s health, and the treatment goals are to improve quality of life and prolong survival (6).

Based on molecular biomarkers and immunohistochemistry (IHC) analyses, breast cancer can be classified into four major subtypes: luminal A, luminal B, HER-2-overexpression, and triple-negative subtypes (7, 8). The luminal A and luminal B subtypes are collectively referred to as Hormone-Receptor Positive HER-2 Negative (HR+HER-2-) (9). HR+ tumors account for approximately 70% of all breast cancers (10). Molecular typing is an important basis for the adjustment of treatment regimens.

For the treatment of HR+HER-2-metastatic breast cancer (HR+HER-2-MBC), international guidelines and expert consensus statements over the past 10 years have recommended endocrine therapy as the first choice in the absence of visceral crisis or endocrine resistance (11–13). However, there has been no prospective study analyzing the difference in efficacy between first-line chemotherapy and first-line endocrine therapy in HR+HER-2-MBC patients. Only a few retrospective studies have shown that chemotherapy is still the most common first-line palliative care for HR+HER-2-MBC (14–17). Studies have shown that endocrine therapy and chemotherapy have similar progression-free survival (PFS) and overall survival (OS) outcomes (18–20). However, there has been no research analyzing the first-line, maintenance, or second-line treatment options for HR+HER-2-MBC.

Clinical specialists are inconsistent in the choice of chemotherapy versus endocrine therapy for the first-line treatment of HR+HER-2-MBC. The decision between chemotherapy and endocrine therapy needs to be made for maintenance therapy and second-line therapy after first-line treatment failure. The above problems suggest the need for optimization of the entire treatment strategy for HR+HER-2-MBC; however, it is difficult to provide clear answers through well-designed clinical trials.

We conducted a retrospective investigation to analyze the clinician selection patterns of first-line, maintenance, and second-line treatment for HR+HER-2-MBC in a real-world setting, as well as the relationship between different patterns and the long-term survival of patients.

## 2 Patients and methods

### 2.1 Patients and data collection

Female patients with MBC diagnosed at Shandong Cancer Hospital and Institute from January 2013 to June 2017 were included in the study.

The purpose of this study was to compare the efficacy of endocrine therapy versus chemotherapy for the treatment of HR+HER-2-MBC as first-line treatment, maintenance treatment, and second-line treatment. The primary endpoint of this study was PFS (first-line/maintenance/second-line treatment), and the secondary endpoints were OS (first-line/maintenance/second-line treatment) and the clinical application status of various treatment modes.

This study mainly analyzed and evaluated the efficacy of endocrine therapy versus chemotherapy for HR+HER-2-MBC in a real-world setting from three perspectives: 1. First-line treatment was either first-line chemotherapy (referred to as the first-line chemotherapy mode) or first-line endocrine therapy (referred to as the first-line endocrine therapy mode). 2. Maintenance treatment after first-line treatment was divided into four different modes. The chemotherapy-endocrine therapy maintenance mode included maintenance endocrine therapy after first-line chemotherapy. The chemotherapy-chemotherapy maintenance mode included maintenance chemotherapy after first-line chemotherapy. The no-maintenance mode refers to no maintenance treatment after first-line chemotherapy. The endocrine therapy-endocrine therapy maintenance mode included endocrine therapy until progression occurred. 3. The first-line/second-line treatment mode was defined as chemotherapy/chemotherapy, chemotherapy/endocrine therapy, endocrine therapy/endocrine therapy, or endocrine therapy/chemotherapy mode. Chemotherapy was used for both first-line and second-line treatment in the chemotherapy/chemotherapy mode. Endocrine therapy was used as first-line and second-line treatment in the late stage (the endocrine therapy/endocrine therapy mode). Chemotherapy was used for first-line treatment and endocrine therapy for second-line treatment in the chemotherapy/endocrine therapy mode. First-line endocrine therapy and second-line chemotherapy were used in the endocrine therapy/chemotherapy mode.

The MBC patients in this study included those with *de novo* stage IV breast cancer and those with recurrent metastatic breast cancer (stage IV). ER positivity and PR positivity were defined as 1% of cells showing positive nuclear staining, and HER-2 negativity was defined as an immunohistochemical score of 0, 1+ or 2+ but a negative fluorescent *in situ* hybridization (FISH) result. The Ki-67 index status was stratified into three categories: low (<15%), intermediate (15–30%), and high (≥30%). Distant metastasis was divided into non-visceral metastasis (including skin, lymph node, and bone metastasis), single visceral metastasis, and multiple visceral metastases (including lung, liver, pericardium, stomach, and other organs) and brain metastasis. Comorbidities included hypertension, coronary heart disease, diabetes, skin disease, cerebral infarction, cervical spondylosis, and other tumors (not affecting the treatment of MBC). PFS was defined as the time from the initiation of initial palliative treatment to progression or death due to the disease. OS was defined as the period from the initiation of initial palliative care to the date of death or the study end date (2021–6). All surviving patients completed and followed up for the last time on the study end date. Tumor progression was evaluated in accordance with the Response Evaluation Criteria in Solid Tumors (RECIST) version 1.1. If imaging or other examination methods could not be used to evaluate the condition (which mostly only occurred in cases of bone metastasis), the initiation of new treatment was defined as progression. In the absence of clear evidence of disease progression (such as bone metastasis), patients who initially chose palliative chemotherapy were switched to endocrine therapy (which was categorized as endocrine maintenance therapy). In the absence of clear evidence of disease progression (such as bone metastasis), patients who received first-line chemotherapy (mostly combination drugs) after achieving stable disease were switched to single-agent chemotherapy or another chemotherapy (which was categorized as chemotherapy maintenance treatment).

Information on baseline patient characteristics, treatment patterns, and disease progression were extracted from patient charts, diagnostic tests, laboratory findings, and clinical notes. Patient demographics (including sex and age), primary tumor information, disease-free interval (DFI), ER status, PR status, HER2 status, Ki-67 index, metastatic location, number of metastatic sites, progression, and treatment data were retrospectively collected by trained data managers. After deidentification, cleaning, and standardization, the data were subjected to professional statistical analysis.

The inclusion criteria were as follows (1): female sex (2); cytologically or histologically confirmed MBC (3); HR+ disease defined as ER-positive and/or PR-positive (4); HER-2- disease according to an immunohistochemical score of 0, 1+ or 2+ but a negative fluorescence in FISH result (5); at least 4 hospital admissions records; and (6) received first-line therapy between January 2013 and June 2017. Patients who did not receive treatment or had other primary malignancies during observation were excluded.

### 2.2 Statistical analysis

Key patient cohorts were stratified by different first-line/second-line treatment modes (chemotherapy/chemotherapy, chemotherapy/endocrine therapy, endocrine therapy/endocrine therapy, or endocrine therapy/chemotherapy mode). Categorical variables were compared using X² tests, and one-way analysis of variance (ANOVA) was used for normally distributed continuous variables, The Wilcoxon rank-sum test was used for skewed continuous data. In this study, the Kaplan–Meier method was used to produce survival curves, and the log-rank method was used to assess the differences between groups. In addition, through Cox proportional hazard model analysis, the prognostic (PFS and OS) differences of patients treated with different treatment modes were analyzed to determine relevant prognostic factors. The prognostic factors included in the model were age, comorbidities, lymph node metastasis, adjuvant endocrine therapy, DFI, symptoms, Ki-67 index, distant metastasis location, and systemic treatment modes. Statistical analysis was performed using SPSS version 25.0 and RStudio (version 1.0.143) with R (version 4.0.0). All reported P values are two-sided, and P values <0.05 were considered statistically significant.

### 2.3 Smote algorithm generates data

There are a number of clinical outcomes have unbalanced proportions, which do not satisfy the balanced endpoint assumption of most Machine-Learning Predictions models (21). In response to this problem, four sampling methods are studied, including down-sampling, upsampling, random oversampling (ROSE) and synthetic minority oversampling technique (SMOTE) (22, 23). The down sampling method down samples most cases during model training, and the upsampling method down samples a few cases. Both approaches are simple, but since the data points created are effectively duplicated and cannot help the prognostic model gain more information, either information is lost or a non-generic decision area is created. SMOTE is an improved sampling method that computes a new synthetic sampling based on the Euclidean distance of the variables. So the synthetic case will have similar attribute values to the existing case, not just a copy like oversampling. Thus, the representation of the minority class in the resulting dataset is increased while reflecting the structure of the original case. Research shows that SMOTE is robust to changes in imbalance ratio under different classifiers (22, 24). In this study, the SMOTE method was adopted to address the problem of group imbalance.

## 3 Results

### 3.1 General characteristics of the patients with HR+HER-2-MBC

The median follow-up period was 43 months (range 7-64.5 months). All HR+HER-2-MBC patients received at least first-line therapy, consisting of at least two cycles, and the clinical efficacy was evaluated (mainly through imaging evaluation, including computed tomography [CT] and magnetic resonance imaging [MRI]). The median age of the patients was 44 years, and only 8.8% of patients were older than 60 years. Approximately 17.6% of patients had high blood pressure, diabetes, or other heart diseases. Seventy-one percent of patients received more than 4 cycles of adjuvant chemotherapy, 64.1% of patients received adjuvant endocrine therapy, and more than half of the patients had a DFI >24 months.

A total of 281 patients completed first-line and second-line treatment and thus were included in the analysis of first-line and second-line treatment modes. The median age of these patients was less than 50 years, and there were few comorbidities. A total of 56.22% of the patients received the chemotherapy/chemotherapy mode, 10.68% of the patients received the endocrine therapy/endocrine therapy mode, 23.49% of the patients received the chemotherapy/endocrine therapy mode, and 9.6% of the patients received the endocrine therapy/chemotherapy mode. There was no statistically significant difference in age, tumor size, pathological type, lymph node metastasis, BMI, Ki-67 index or DFI between the groups of patients treated with different systemic treatment modes. The patients in the four treatment mode groups had significant differences in Karnofsky’s performance status (KPS), clinical symptoms, adjuvant endocrine therapy, and distant metastasis (**Table 1**).

**Table 1:** Characteristics of HR+HER-2-MBC patients with different first-line/second-line treatment modes.

Characteristics	ALL N=281	chemotherapy/chemotherapy mode N=158n (%)	endocrine/endocrine mode N=30n (%)	chemotherapy/endocrine mode N=66n (%)	endocrine/chemotherapy mode N=27n (%)	P-value
Age at initiation of treatment						
Median (minimum; maximum)	44 (23-72)	44 (23-72)	42 (29-69)	44.5 (30-70)	42 (26-63)	0.890
<60	258 (91.8)	143 (90.5)	28 (93.3)	62 (93.9)	25 (92.6)	0.914
≥60	23 (8.2)	15 (9.5)	2 (6.7)	4 (6.1)	2 (7.4)	
Comorbidity						0.261
Yes	77 (27.4)	46 (29.1)	11 (36.7)	16 (24.2)	4 (14.8)	
No	204 (72.6)	112 (70.9)	19 (63.3)	50 (75.8)	23 (85.2)	
BMI						0.353
≤28	224 (79.7)	128 (81.0)	25 (83.3)	53 (80.3)	18 (66.7)	
>28	57 (20.3)	30 (19.0)	5 (16.7)	66 (19.7)	9 (33.3)	
KPS						0.025
≤80	108 (38.4)	72 (45.6)	6 (20.0)	22 (33.3)	8 (29.6)	
>80	173 (61.6)	86 (54.4)	24 (80.0)	44 (66.7)	19 (70.4)	
Primary tumor stage						0.969
≤5cm	157 (88.2)	74 (87.1)	23 (92.0)	42 (87.5)	18 (90.0)	
>5cm	21 (11.8)	11 (12.9)	2 (8.0)	6 (12.5)	2 (10.0)	
Primary lymph node stage						0.790
Node negative	58 (26.1)	29 (25.0)	9 (33.3)	15 (26.8)	5 (21.7)	
Node positive	164 (73.9)	87 (75.0)	18 (66.7)	41 (73.2)	18 (78.3)	
Pathological type						0.836
Invasive ductal carcinoma	226 (82.8)	124 (80.5)	24 (82.8)	55 (84.6)	23 (92.0)	
Invasive lobular carcinoma	19 (7.0)	12 (7.8)	3 (10.3)	3 (4.6)	1 (4.0)	
Others	28 (10.3)	18 (11.7)	2 (6.9)	7 (10.8)	1 (4.0)	
Her-2 expression intensity						0.842
0	145 (52.5)	84 (53.8)	15 (51.7)	34 (53.1)	12 (44.4)	
1+/2+^1^	131 (47.5)	72 (46.2)	14 (48.3)	30 (46.9)	15 (55.6)	
KI-67						0.715
<15%	102 (36.3)	62 (39.2)	13 (43.3)	19 (28.8)	8 (29.6)	
15-30%	53 (18.9)	28 (17.7)	6 (20.0)	14 (21.2)	5 (18.5)	
≥30%	126 (44.8)	68 (43.0)	11 (36.7)	33 (50.0)	14 (51.9)	
Symptom						<0.001
Yes	167 (59.4)	110 (69.6)	11 (36.7)	36 (54.5)	10 (37.0)	
No	114 (40.6)	48 (30.4)	19 (63.3)	30 (45.5)	17 (63.0)	
Adjuvant chemotherapy						0.053
≥4 cycles	196 (69.8)	101 (63.9)	22 (73.3)	53 (80.3)	20 (74.1)	
<4 cycles	75 (26.7)	51 (32.3)	8 (26.7)	12 (18.2)	4 (14.8)	
unspecified	10 (3.6)	6 (3.8)	0 (0.0)	1 (1.5)	3 (11.1)	
Adjuvant endocrine therapy						0.043
Yes	180 (64.1)	90 (57.0)	23 (76.7)	48 (72.7)	19 (70.4)	
No	101 (35.9)	68 (43.0)	7 (23.3)	18 (27.3)	8 (29.6)	
Disease-free intermission						0.644
≤24	103 (36.7)	61 (38.6)	14 (46.7)	20 (30.3)	8 (29.6)	
≤60	101 (35.9)	55 (34.8)	10 (33.3)	24 (36.4)	12 (44.4)	
>60	77 (27.4)	42 (26.6)	6 (20.0)	22 (33.3)	7 (25.9)	
Initial metastatic sites						0.015
No visceral metastasis	155 (55.2)	78 (49.4)	26 (86.7)	35 (53.0)	16 (59.3)	
Single visceral metastases	96 (34.2)	58 (36.7)	3 (10.0)	27 (40.9)	8 (29.6)	
Multiple visceral metastases	18 (6.4)	14 (8.9)	0 (0.0)	3 (4.5)	1 (3.7)	
Brain metastases	12 (4.3)	8 (5.1)	1 (3.3)	1 (1.5)	2 (7.4)	

Characteristics of patients with Hormone-Receptor positive HER-2 negative Metastatic Breast Cancer (HR+HER-2-MBC), according to first-line/second-line treatment mode, and 281 patients entered first-line/second-line treatment mode. 1.Her-2 expression intensity 1+/2+FISH negative.

There was no difference in the distribution of low (<15%)/intermediate (15–30%)/high (≥30%) KI-67 index between the first-line treatment mode patients and the same results were obtained for the maintenance treatment mode and first-line/second-line mode patients (**Table 2**).

**Table 2:** Distribution of the expression of Ki-67 in different treatment modes.

KI-67	<15%	15-30%	≥30%	P-value
First-line treatment modes				P=0.975
First-line endocrine mode	23(20.5%)	11(19.3%)	27(19.6%)	
First-line chemotherapy	89(79.5%)	46(80.7%)	111(80.4%)	
Maintenance treatment modes				P=0.973
Chemotherapy-endocrine maintenance mode	35(31.3%)	20(35.1%)	49(35.5%)	
Chemotherapy-chemotherapy Maintenance mode	8(7.1%)	2(3.5%)	7(5.1%)	
No maintenance mode	46(41.1%)	24(42.1%)	55(39.9%)	
Endocrine-endocrine maintenance mode	23(20.5%)	11(19.3%)	27(19.6%)	
First-line/second-line treatment modes				P=0.715
Chemotherapy/chemotherapy	62(60.8%)	28(52.8%)	68(54.0%)	
Endocrine/endocrine	13(12.7%)	6(11.3%)	11(8.7%)	
Chemotherapy/endocrine	19(18.6%)	14(26.4%)	33(26.2%)	
Endocrine/chemotherapy	4(7.8%)	5(9.4%)	14(11.1%)	

### 3.2 Overview of systemic treatment modes for HR+HER-2-MBC patients

**Figure 1:**
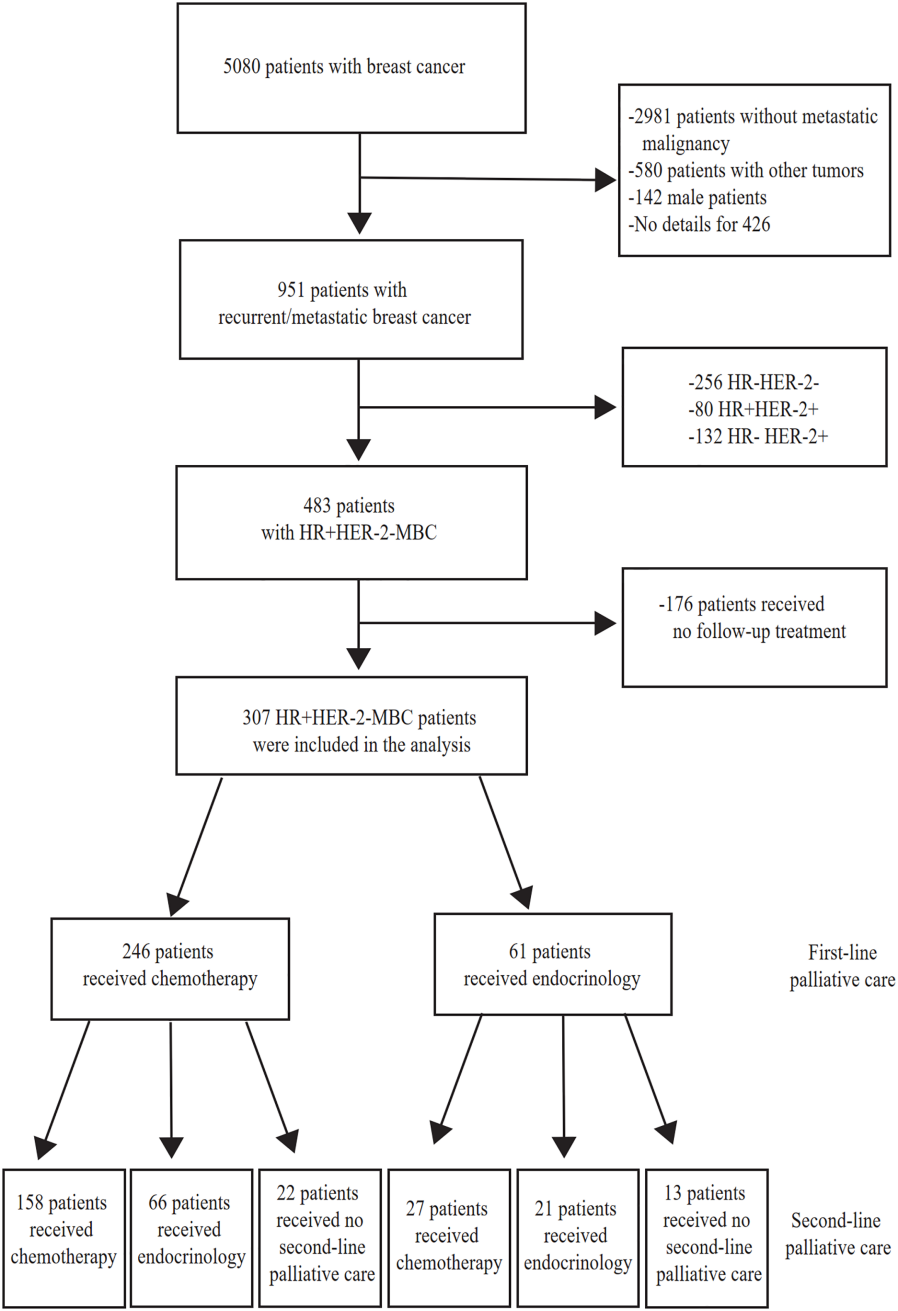
Study flow chart. MBC, Metastatic Breast Cancer. HR+, Hormone-receptor positive. HR-, Hormone-receptor negative. HER-2-, HER-2 negative. HER-2+ HER-2 is positive.

Of the 951 patients with MBC, we included 307 HR+HER-2-MBC patients for analysis. A total of 256 patients had HR-HER-2-MBC, 80 patients had HR+HER-2+MBC, and 132 patients had HR-HER-2+MBC; 176 patients without follow-up data were excluded. Of the 307 patients with HR+HER-2-MBC who were included, 246 (80.13%) were treated with first-line chemotherapy, and 61 (19.87%) were treated with first-line endocrine therapy (**Figure 1**).

Among the 246 patients who received chemotherapy as first-line treatment, 121 achieved stable disease and entered the maintenance treatment stage. Among them, the chemotherapy-endocrine therapy maintenance mode was used in 104 patients (85.95%), and the chemotherapy-chemotherapy maintenance mode was used in 17 patients (9.34%). Another 125 patients did not receive maintenance treatment, for example, because interruption of treatment after first-line chemotherapy was effective or because they were switched to second-line treatment after repeat disease progression.

Among the 281 HR+HER-2-MBC patients who received at least second-line treatment, 158 patients (56.23%) received the chemotherapy/chemotherapy mode, and 30 patients (10.68%) received the endocrine therapy/endocrine therapy mode. Sixty-six patients (23.49%) received the chemotherapy/endocrine therapy mode. Twenty-seven patients (9.6%) received the endocrine therapy/chemotherapy mode (**Table 1**).

### 3.3 The relationship between different systemic treatment modes and survival in patients with HR+HER-2-MBC

#### 3.3.1 Survival analysis of the two first-line treatment modes

##### 3.3.1.1 *Univariate analysis*

**Figure 2:**
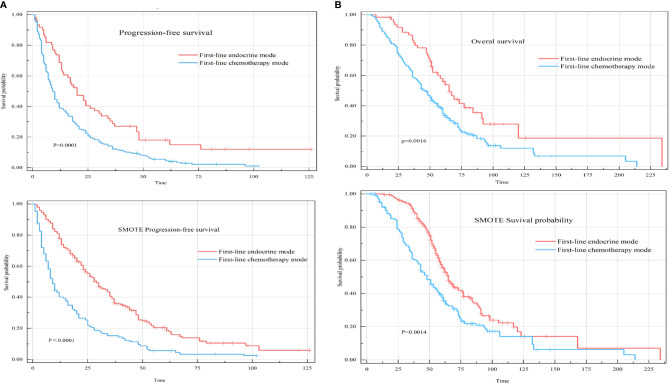
Progression-free survival (PFS) and overall survival (OS) of two different first-line treatment modes for hormone receptor-positive HER-2-negative metastatic breast cancer (HR+HER-2-MBC) patients. **(A)** Kaplan–Meier curve of PFS in first-line treatment modes. **(B)** Kaplan–Meier curve of OS in first-line treatment modes.

All included HR+HER-2-MBC patients were included in the univariate analysis of the first-line therapy mode to determine differences in PFS and OS. The results showed that the median PFS of patients who received the first-line endocrine therapy mode was 20 months (95% confidence interval [CI], 14-30), and the median PFS of patients who received the first-line chemotherapy mode was 10 months (95% CI: 8-12) (P<0.0001) (**Figure 2A**). The median OS of patients who received the first-line endocrine therapy mode was 65 months (95% CI: 56-92), while the median OS of patients who received the first-line chemotherapy mode was 48 months (95% CI: 43-55) (P=0.0016) (**Figure 2B**).

##### 3.3.1.2 *Multivariate analysis*

**Figure 3:**
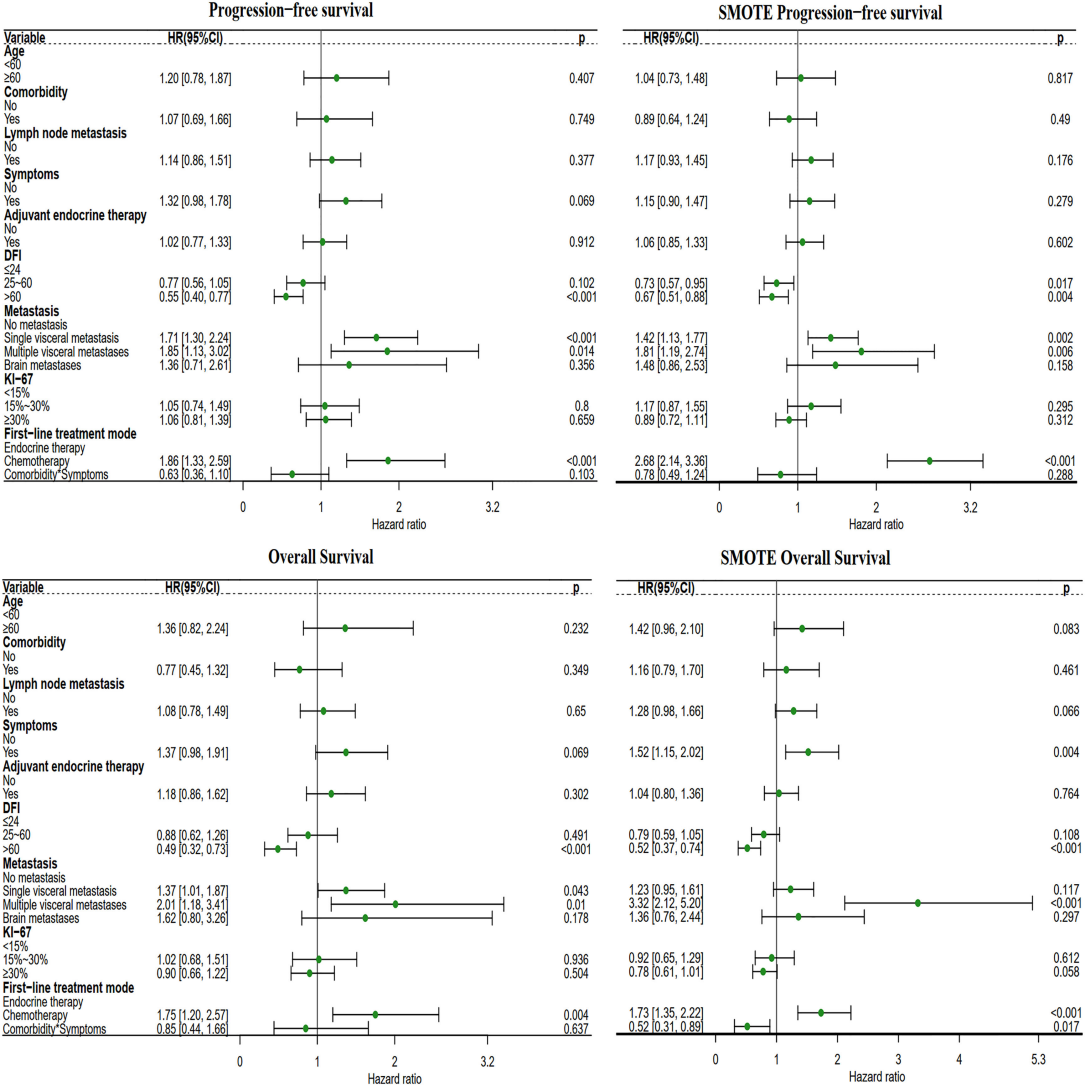
Forest plot of the two different first-line treatment modes. Correcting confounding factors that may affect prognosis in addition to treatment. Age, age≥60 years. Comorbidities, Comorbidities included hypertension, coronary heart disease, diabetes, skin disease, cerebral infarction, cervical spondylosis, and other tumors (not affecting the treatment of MBC). Lymph node metastasis, lymph node metastasis ≥1. Symptom, Positive clinical symptoms. In adjuvant endocrine therapy, the patient has undergone adjuvant endocrine therapy, including standardized and nonstandard adjuvant endocrine therapy. First-line chemotherapy mode, compared with the first-line endocrine therapy mode. DFI, Disease-Free Interval. Distant metastasis, distant metastasis includes non-visceral metastasis (skin, lymph node, bone metastasis, etc.) and single/multiple visceral metastases (including lung, liver, stomach, kidney metastasis, etc.).

After adjusting for known prognostic factors, including age, comorbidities, lymph node metastasis, adjuvant endocrine therapy, DFI, symptoms, Ki-67 index, distant metastasis location, and systemic treatment modes., patients who received first-line chemotherapy had poorer PFS and OS than patients who received first-line endocrine therapy. Compared with patients who received the first-line endocrine therapy mode, patients who received the first-line chemotherapy mode had a PFS hazard ratio (HR) of 1.86 (95% CI: 1.33-2.59, P<0.001) and an OS HR of 1.75 (95% CI: 1.20-2.87, P=0.004) (**Figure 3**).

#### 3.3.2 Survival analysis of the four maintenance treatment modes

##### 3.3.2.1 *Univariate analysis*

**Figure 4:**
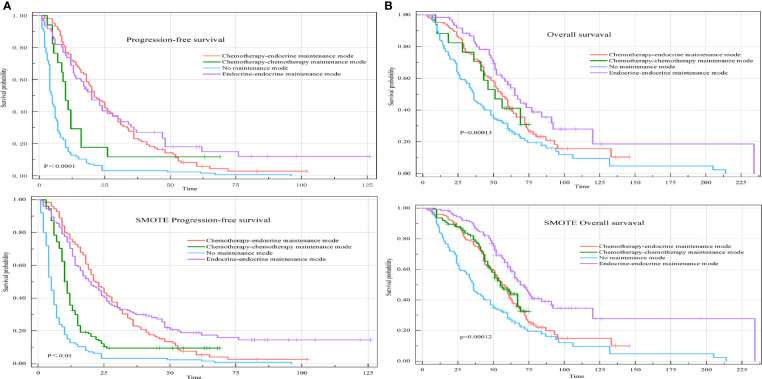
PFS and OS of HR+HER-2-MBC patients were treated with four different maintenance treatment modes. **(A)** Kaplan–Meier curve of PFS in maintenance treatment modes. **(B)** Kaplan–Meier curve of OS in maintenance treatment modes.

The median PFS of the chemotherapy-endocrine therapy maintenance mode group was 22.50 months (95% CI: 19.5-28). The median PFS of the chemotherapy-chemotherapy maintenance mode group was 10 months (95% CI: 8.5-16). The median PFS of the no-maintenance mode group was 4.75 months (95% CI: 4-6). The median PFS of the endocrine therapy-endocrine therapy maintenance mode group was 20 months (95% CI: 14-30) (P<0.0001) (**Figure 4A**).

The median OS of the chemotherapy-endocrine therapy maintenance mode group was 58 months (95% CI: 51-66). The median OS of the chemotherapy-chemotherapy maintenance mode group was 51 months (95% CI: 33-68). The median OS of the no-maintenance mode group was 36 months (95% CI: 33-48). The median OS of the endocrine therapy-endocrine therapy maintenance mode group was 65 months (95% CI: 56-92) (P=0.00013) (**Figure 4B**).

##### 3.3.2.2 *Multivariate analysis*

**Figure 5:**
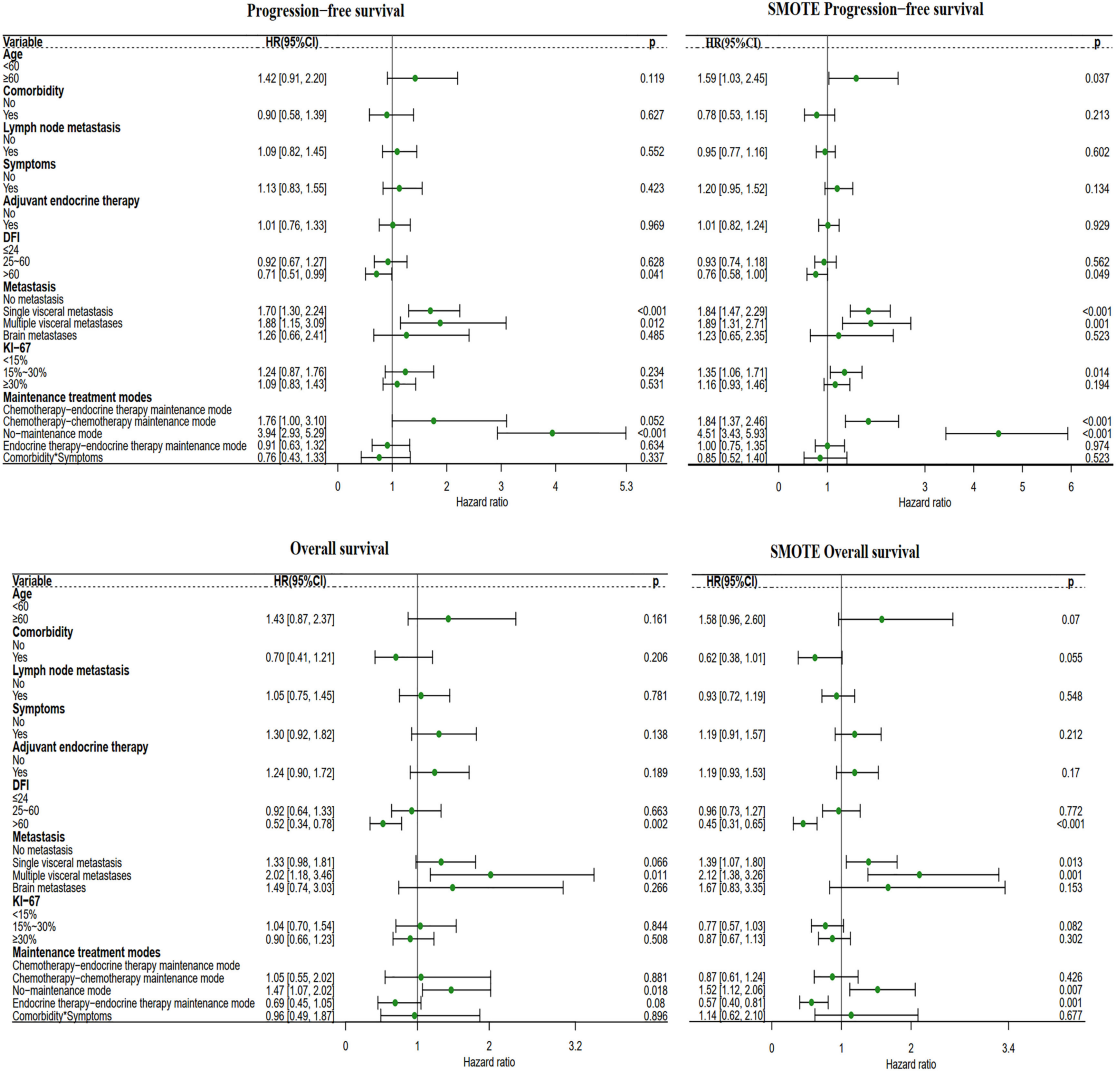
Forest plot of the four different maintenance treatment modes. Maintenance treatment modes and maintenance treatment modes included the chemotherapy-endocrine therapy maintenance mode, chemotherapy-chemotherapy maintenance mode, the no-maintenance mode, and endocrine therapy-endocrine therapy maintenance mode. In this analysis, the other three modes are compared with the chemotherapy-endocrine therapy maintenance mode.

Compared with patients in the chemotherapy-endocrine therapy maintenance mode group, patients in the endocrine therapy-endocrine therapy maintenance mode group had a PFS HR of 0.91 (95% CI: 0.63-1.32, P=0.634) and an OS HR of 0.69 (95% CI: 0.45-1.05, P=0.08). The chemotherapy-chemotherapy maintenance mode group and the no-maintenance mode group were associated with a poor prognosis (**Figure 5**).

#### 3.3.3 Survival analysis of the four first-line/second-line treatment modes

##### 3.3.3.1 *Univariate analysis*

**Figure 6:**
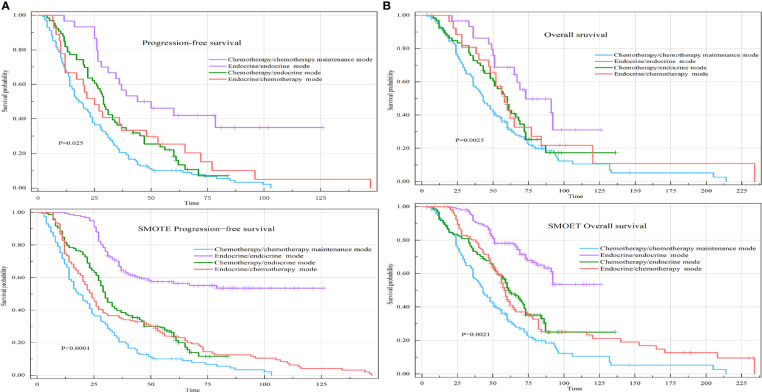
PFS and OS of the four different first-line/second-line treatment modes. **(A)** Kaplan–Meier curve of PFS in the four different first-line/second-line treatment modes. **(B)** Kaplan–Meier curve of OS in the four different first-line/second-line treatment modes.

The median PFS (PFS=PFS1+PFS2) of the chemotherapy/chemotherapy mode group was 20 months (95% CI: 16-24). The median PFS of the endocrine therapy/endocrine therapy mode group was 34.5 months (95% CI: 26.5-60). The median PFS of the chemotherapy/endocrine therapy mode group was 28.5 months (95% CI: 22-33). The median PFS of the endocrine therapy/chemotherapy mode group was 25 months (95% CI: 17-53) (P=0.025) (**Figure 6A**).

The median OS of the chemotherapy/chemotherapy mode group was 43 months (95% CI: 37-51). The median OS of the endocrine therapy/endocrine therapy mode group was 73 months (95% CI: 46-99). The median OS of the chemotherapy/endocrine therapy mode group was 60 months (95% CI: 50-69). The median OS of the endocrine therapy/chemotherapy mode group was 58 months (95% CI: 48-84) (P=0.0025) (**Figure 6B**).

##### 3.3.3.2 *Multivariate analysis*

**Figure 7:**
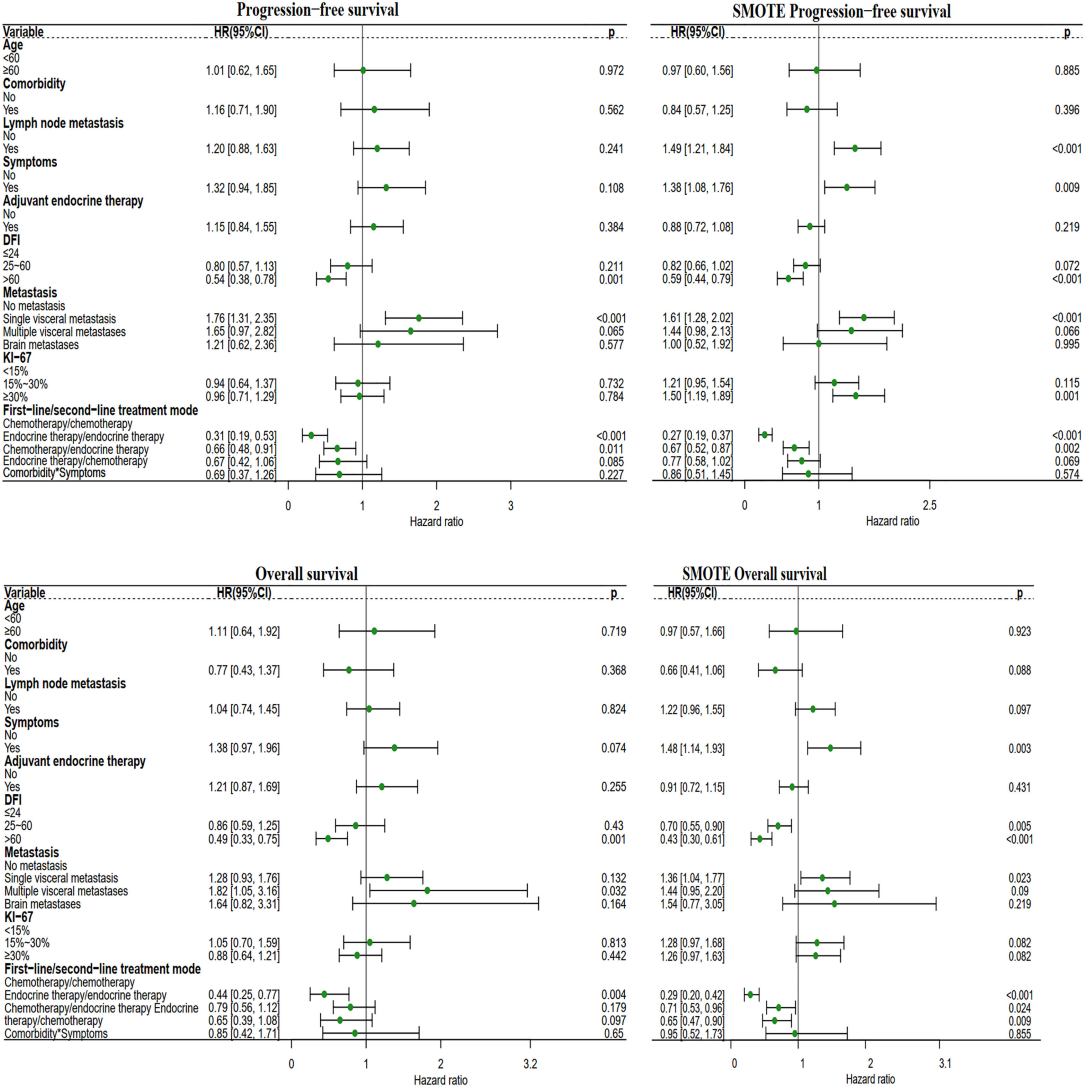
Forest plot of the four different first-line/second-line treatment modes. The first-line/second-line treatment mode, includes the chemotherapy/chemotherapy mode, endocrine therapy/endocrine therapy mode, chemotherapy/endocrine therapy mode, and endocrine therapy/chemotherapy mode. In this analysis, the other three modes were compared with the chemotherapy/chemotherapy mode.

Compared with patients receiving the chemotherapy/chemotherapy mode, patients receiving the endocrine therapy/endocrine therapy mode had a PFS HR of 0.31 (95% CI: 0.19-0.53, P<0.001) and an OS HR of 0.44 (95% CI: 0.25-0.77, P=0.004). Endocrine therapy significantly reduced the risk of survival independent of other prognostic factors. DFI> 60 months significantly reduced the survival risk. Obvious clinical symptoms and multiple visceral metastases were all related to a poor prognosis. After SMOTE, we found that lymph node metastases, clinical symptoms, visceral metastases, and Ki-67 index ≥ 30% were associated with poorer prognosis (**Figure 7**).

### 3.4 The relationship between the expression of Ki-67 and the prognosis of HR+HER-2-MBC

**Figure 8:**
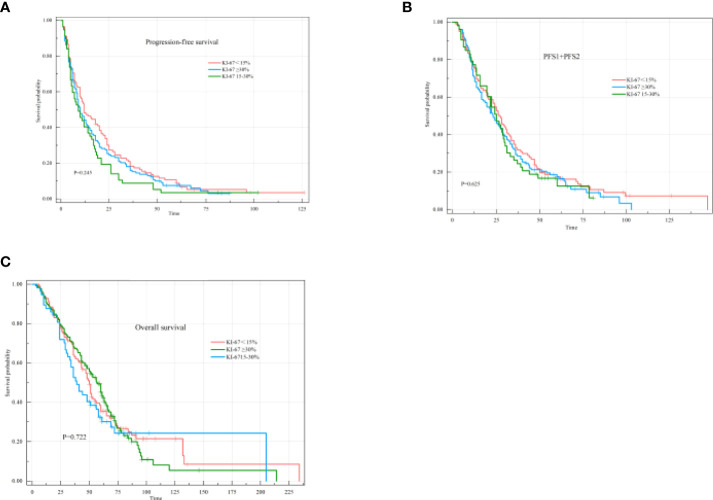
PFS1, PFS1+PFS2, and OS of different states according to the KI-67 index. **(A)** Kaplan–Meier curve of PFS1 in different states according to the KI-67 index. **(B)** Kaplan–Meier curve of PFS1+PFS2 in different states according to the KI-67 index. **(C)** Kaplan–Meier curve of OS in different states according to the KI-67 index.

The Ki-67 index was stratified into three categories: low (<15%), intermediate (15–30%), and high (≥30%). The median PFS1 was 12 months (95% CI: 6.93-17.07) for patients with a KI-67 index<15%, 10 months (95% CI: 5.89-14.11) for patients with a Ki-67 index 15-30%, and 10 months (95% CI: 7.31-12.69) for patients with a KI-67 index≥30% (P=0.245), with no significant differences observed among the groups (**Figure 8A**). The median PFS1+PFS2 was 26 months (95% CI: 21.61-31.39) for patients with a KI-67 index<15%, 25 months (95% CI: 19.46-30.54) for patients with a Ki-67 index 15-30%, and 23 months (95% CI: 17.84-28.16) for patients with a KI-67 index≥30% (P=0.625), with no significant differences observed among the groups (**Figure 8B**). The median OS was 51 months (95% CI: 47.24-54.77) for patients with a KI-67 index<15%, 41 months (95% CI: 28.68-53.32) for patients with a Ki-67 index of 15-30%, and 60 months (95% CI: 52.97-67.03) for patients with a KI-67 index≥30% (P=0.722), also with no significant differences observed among the groups (**Figure 8C**).

## 4 Discussion

Compared with other types of breast cancer, HR+HER-2-MBC progresses slower and is relatively insensitive to chemotherapy. However, HR+HER-2-MBC patients have a unique treatment option—endocrine therapy (25). Treatment plan formulation needs to consider various factors to determine whether chemotherapy or endocrine therapy is more appropriate. These factors include patient age, molecular pathological characteristics, tumor burden, speed of disease development, physical symptoms, previous treatment(s), DFI, and comorbidities. Endocrine therapy and chemotherapy can be used as a systemic treatment at various stages, including first-line treatment, maintenance treatment after effective first-line treatment, and second-line treatment after first-line treatment failure.

Although international guidelines have recommended endocrine therapy as the first choice for HR+HER-2-MBC for the past 10 years and provide clear limitations regarding its use for first-line chemotherapy (11, 12, 26, 27), in clinical practice, most doctors disregard these recommendations and prefer to choose chemotherapy. In addition, both chemotherapy and endocrine therapy for maintenance treatment after chemotherapy are effective, although there are clinical variations. Determining how to optimize the choice of treatment to maximize survival and improve quality of life is a topic worthy of further consideration.

A retrospective study of HR+HER-2-MBC by Gupta et al. found that 53% of patients received advanced first-line chemotherapy, and 47% received advanced first-line endocrine therapy (15). Caswell-Jin et al. showed that 43% of patients received first-line chemotherapy, and 67% received first-line endocrine therapy (16). We evaluated the systemic treatment options for HR+HER-2-MBC at a single hospital over the past 6 years and found that more than four-fifths of the patients received chemotherapy as first-line therapy, while less than 20% of the patients received endocrine therapy as first-line therapy. According to the available guidelines during the study period, most patients should have received endocrine therapy, not chemotherapy. This retrospective study was performed before the development of the oncology department at our hospital, which implies that before the Department of Breast Oncology became an independent department, medical oncologists were reluctant to accept the recommendation of endocrine therapy for patients with HR+HER-2-MBC.

Lobbezoo et al. reported median PFS values for first-line chemotherapy and first-line endocrine therapy of 5.3 months (95% CI: 4.2-6.2) and 13.3 months (95% CI: 11.3-15.5), respectively, and median OS values of 16.1 and 36.9 months, respectively. After adjusting for prognostic factors, first-line chemotherapy was associated with poorer PFS and OS outcomes (18). Other studies have also shown that first-line endocrine therapy yields significantly better PFS and OS outcomes than first-line chemotherapy (19, 20, 28). These results are similar to the results obtained in our study. However, the PFS and OS results of the overall population in our study were better than those reported in other studies, which may be related to the younger age and fewer comorbidities of this population of Chinese patients with breast cancer. Compared with breast cancer patients in Western countries such as Europe and the United States, the age of onset of breast cancer in China is at least 10 years younger, with a median age of approximately 45 years. In addition, other features such as lymph node metastases are common in early-stage Chinese patients, along with a high histological grade and a low percentage of HR positivity observed among these patients (29, 30).

Our study also analyzed the maintenance treatment options after first-line chemotherapy. A total of 121 patients (49.19%) had stable disease after first-line chemotherapy and entered the maintenance treatment stage. Of these, 104 patients (85.95%) received chemotherapy-endocrine therapy maintenance treatment. However, 17 patients (9.34%) received chemotherapy-chemotherapy maintenance treatment. Both the median PFS time and median OS time were significantly different between these two groups. As for maintenance treatment, endocrine therapy was found to be confer better outcomes than chemotherapy. We also found that the endocrine therapy-endocrine therapy maintenance mode produced the greatest survival benefit. No maintenance treatment had poor efficacy. The no-maintenance mode group included patients who experienced treatment interruption after first-line chemotherapy and started second-line treatment after repeat progression, patients who were switched directly to second-line treatment after first-line chemotherapy failure, and patients who discontinued treatment after first-line chemotherapy failure. The relationship between the maintenance treatment mode and prognosis has not yet been reported in the literature.

Finally, we analyzed the impact of the four different combinations of systemic first-line and second-line treatment modes on survival. The results showed that the endocrine therapy/endocrine therapy mode achieved superior PFS and OS outcomes compared to the chemotherapy/chemotherapy mode, chemotherapy/endocrine therapy mode, and endocrine therapy/chemotherapy mode, and this difference was statistically significant. Interestingly, the chemotherapy/endocrine therapy mode achieved better PFS and OS outcomes than the endocrine therapy/chemotherapy mode. The reason for this result may be that patients with primary endocrine resistance are more likely to receive second-line chemotherapy. Among all the first-line/second-line treatment modes analyze, the endocrine therapy/endocrine therapy mode had the best effect independent of other possible prognostic factors. Although the chemotherapy/chemotherapy mode group included a portion of the population who received endocrine therapy maintenance treatment after first-line chemotherapy, this group exhibited the poorest outcomes.

A study from Italy showed that fulvestrant (500 mg) was effective in patients receiving treatment both upon disease progression and as maintenance therapy. The median OS time of the whole population was 26.8 months, ranging from 32.4 months for patients receiving first-line treatment to 22.0 and 13.7 months for those receiving second-line and subsequent-line treatment, respectively. Good outcomes are associated with endocrine sensitivity (31). Our study also showed that endocrine therapy is superior to chemotherapy as first-line, second-line, and maintenance therapy. Regarding the types of endocrine therapy, AI and fulvestrant are the main ones. Interestingly, since fulvestrant is an injection, it is not as widely used in the maintenance phase.

These results indicate that endocrine therapy plays an important role in the treatment of HR+MBC, regardless of whether it is administered as first-line treatment, maintenance therapy, or second-line treatment. Oncologists should take this into consideration when designing treatment plans to prolong the survival of patients and improve their quality of life.

The cut-off value of the KI-67 index has not yet been uniformly defined (32). In this study, the KI-67 index was divided into three categories according to expert consensus over the years during the study period: low (<15%), intermediate (15–30%), and high (≥30%) (33–37). According to this classification, the distribution of the KI-67 indexes did not differ among the patients receiving different systemic treatment modalities. There was no difference in the effect of the KI-67 index on survival, which may be due to the large distribution gap between patients who chose chemotherapy and endocrine therapy and the high proportion of patients receiving chemotherapy. However, after SMOTE, we found that KI-67 index ≥ 30% had a poor prognosis. Further analysis found that 80.58% of patients with KI-67 index ≥30% chose first-line chemotherapy. In the original data analysis, we did not see an effect of KI-67 on survival. However, after SMOTE, adverse effects of KI-67 on PFS were seen. It is known that the sample size contributed to this bias. However, only in the first-line/second-line treatment mode, the effect of KI-67 index on PFS1+PFS2 was statistically different. A high KI-67 index also had a bad effect on OS, but it was not statistically significant. The expression of various molecules in breast cancer is highly heterogeneous, especially the KI-67 index. The KI-67 index after neoadjuvant chemotherapy may be lower than that before treatment (34). In this study, the KI-67 index was based on the results of pathological biopsy before advanced treatment. Treatment can affect the expression of KI-67 index, and the effect of treatment on survival is more important than KI-67 index. Studies have shown that the expression of Ki-67 is closely related to tumor cell proliferation and growth and is often assessed as a proliferation marker (38). A high KI-67 index is often associated with a good response to chemotherapy (39). Most of the patients in this study chose chemotherapy. After SMOTE balances the difference in sample size, the impact of KI-67 index on prognosis is prominent.

This was a retrospective analysis of real-world data with some limitations. First, not all data provided by doctors were transferred into the medical records. In addition, despite efforts to adjust the results based on clinically meaningful and relevant patient characteristics, some confounding factors may not have been considered in the adjustment of baseline characteristics. Only a good randomized controlled trial can avoid discrepancies caused by unrecognized factors. In addition, selection bias may exist, and a small number of patients from the outpatient clinic may have been overlooked.

Since the advent of CDK4/6 inhibitors in 2016, an increasing number of studies have proven that the effects of an aromatase inhibitor (AI) + CDK4/6 inhibitors are significantly better than those of AI monotherapy, significantly prolonging PFS (13, 40). Studies have shown that compared with fulvestrant alone, fulvestrant combined with CDK4/6 inhibitors has significant benefits for PFS (41–43). As these new treatments change the outcome of HR+HER-2-MBC, they are expected to also change the systemic treatment model. As of the follow-up endpoint of June 2021, CDK4/6 inhibitors are still not covered by medical insurance in China, which means that patients must pay out-of-pocket for these medications, and most patients cannot afford such a large medical expense, so the accessibility of CDK4/6 inhibitors in China must be improved. In addition, some patients who receive CDK4/6 inhibitors cannot tolerate their serious adverse reactions, such as diarrhea, and bone marrow suppression. This study aims to provide optimal treatment options for these patients.

## 5 Conclusion

In conclusion, the real-world data presented herein provide a good overview of the treatment of HR+HER-2-MBC. Endocrine therapy plays an important role in the entire treatment process of HR+HER-2-MBC patients. The continuous advent of new drugs will continue to change clinical decision-making. Whether chemotherapy or endocrine therapy is optimal and the choice of different chemotherapeutic drugs, endocrine drugs, and targeted drugs are all worthy of consideration and further exploration. Therefore, continuing to collect real-world data is essential for evaluating the treatment of MBC and optimizing treatment strategies.

## Data Availability Statement

The raw data supporting the conclusions of this article will be made available by the authors, without undue reservation.

## Ethics Statement

The studies involving human participants were reviewed and approved by the ethics committee of Shandong Cancer Hospital and Institute, Jinan, Shandong, People’s Republic of China. Written informed consent for participation was not required for this study in accordance with the national legislation and the institutional requirements.

## Author Contributions

SF and BB designed the study and revised the article. QYL performed the study, processed the data analysis and interpretation, and drafted the manuscript. JQ, QRL, and YM collected data and processed the data analysis. LS supervised the research and provided financial support for the project. All authors contributed to the article and approved the submitted version.

## Funding

This work is supported by Shandong Cancer Hospital and Institute. No commercial funding was received.

## Conflict of Interest

The authors declare that the research was conducted in the absence of any commercial or financial relationships that could be construed as a potential conflict of interest.

## Publisher’s Note

All claims expressed in this article are solely those of the authors and do not necessarily represent those of their affiliated organizations, or those of the publisher, the editors and the reviewers. Any product that may be evaluated in this article, or claim that may be made by its manufacturer, is not guaranteed or endorsed by the publisher.
